# Expression of Concern: SH2 Modified STAT1 Induces HLA-I Expression and Improves IFN-γ Signaling in IFN-α Resistant HCV Replicon Cells

**DOI:** 10.1371/journal.pone.0266497

**Published:** 2022-03-31

**Authors:** The PLOS ONE Editors

After this article [1] was published, concerns were raised about western blots in Figs 2, 5 and 7, and microscopy images in Figs 1C, 6 and 8.

Specifically:

- In Fig 1C in this article, the GR17-1 IFN-γ (-) panel appears similar to the 15–3 Cells IFN-α (+) panel in Fig 7a in [2], and the GR17-1 IFN-γ (+) panel appears similar to the IRF9 panel in Fig 7A in [2].
- In Fig 2 in this article, the backgrounds of lanes one and two in the p-Jak2 blot appear similar to each other.
- In Fig 5 in this article, lanes three, four, six, and seven in the GR17-1 blot appear similar to each other.
- In Fig 6A in this article, the differential interference contrast image for IFN-γ (-) appears similar to the S9-13 IFN-γ (-) STAT1-CC-GFP panel in Fig 6B with 90° rotation. The differential interference contrast image in Fig 6A for IFN-γ (+) appears similar to the S9-13 IFN-γ (+) STAT1-GFP panel in Fig 6B with 90° rotation.
- In Fig 6B in this article, the GR17-1 IFN-γ (+) STAT1-GFP panel appears similar to the GR17-1 IFN-γ (-) STAT1-CC-GFP panel.
- In Fig 7 in this article, the GADPH blot appears similar to the HCV blot with an additional band in the final lane for the GADPH blot.
- In Fig 8 in this article, the S-Huh7 panel appears similar to the Huh-7 Cells IFN-α (-) panel in Fig 7A in [2] and to the Huh-7 Cells IFN-α (+) panel in Fig 7A in [2].

Follow-up on these issues is ongoing; in the meantime, the *PLOS ONE* Editors issue this Expression of Concern.
